# Beware of the commercialization of human cells and tissues: situation in the European Union

**DOI:** 10.1007/s10561-012-9323-3

**Published:** 2012-06-21

**Authors:** Jean-Paul Pirnay, Alain Vanderkelen, Nadine Ectors, Christian Delloye, Denis Dufrane, Etienne Baudoux, Michel Van Brussel, Michael P. Casaer, Daniel De Vos, Jean-Pierre Draye, Thomas Rose, Serge Jennes, Pierre Neirinckx, Geert Laire, Martin Zizi, Gilbert Verbeken

**Affiliations:** 1 Human Cell and Tissue Banks, Laboratory for Molecular and Cellular Technology, Burn Wound Centre, Queen Astrid Military Hospital, Bruynstraat 1, 1120 Brussel, Belgium; 2 Tissue Banks, University Hospitals Leuven, KU Leuven, Leuven, Belgium; 3 Orthopaedic and Trauma Surgery Department, Cliniques Universitaires St-Luc, Université Catholique de Louvain, Brussel, Belgium; 4 Center of Tissular/Cellular Therapy, Cliniques Universitaires St-Luc, Université Catholique de Louvain, Brussel, Belgium; 5 Laboratory of Cellular and Gene Therapy, University Hospital Liège, Liège, Belgium; 6 Burn Centre, University Hospitals Leuven, Leuven, Belgium; 7 Burn Wound Centre, Queen Astrid Military Hospital, Brussel, Belgium; 8 Queen Astrid Military Hospital, Brussel, Belgium; 9 Medical Component, Ministry of Defense, Brussel, Belgium; 10 Well Being Department, Queen Astrid Military Hospital, Brussel, Belgium; 11 Department of Physiology, Free University Brussels, Brussel, Belgium

**Keywords:** Human cells and tissues, European Union, Directive, Quality and safety, Advanced therapy medicinal products, Globalization

## Abstract

With this analysis we would like to raise some issues that emerge as a result of recent evolutions in the burgeoning field of human cells, tissues, and cellular and tissue-based product (HCT/P) transplantation, and this in the light of the current EU regulatory framework. This paper is intended as an open letter addressed to the EU policy makers, who will be charged with the review and revision of the current legislation. We propose some urgent corrections or additions to cope with the rapid advances in biomedical science, an extensive commercialization of HCT/Ps, and the growing expectation of the general public regarding the ethical use of altruistically donated cells and tissues. Without a sound wake-up call, the diverging interests of this newly established ‘healthcare’ industry and the wellbeing of humanity will likely lead to totally unacceptable situations, like some of which we are reporting here.

## Incidents

Since the late 90s, the field of human cells, tissues, and cellular and tissue-based product (HCT/P) transplantation is booming and the market value of some replacement parts such as bone and heart valves has been identified as very attractive.

However, some ethical and safety scandals emerged such as non-consented procurement, inadequate testing, inaccurate or false donor files, irresponsible allocations and illegal trafficking of HCT/Ps. Hearings, lawsuits, convictions, resignations and the shutdown of Tissue Establishments (TEs) followed. Mediatized cases such as the ‘France Hypophyse scandal’ (Spurgeon 2008), the ‘New York body-snatching ring’ (Waltz 2006) and the ‘Alder Hey organ retention scandal’ (Redfern et al. 2001) drew public attention and questioned the adequacy of the regulatory framework that governed the HCT/P industry (Collins 2001).

## EU legislation

In 2004 the European Commission (EC) issued the EU Cells and Tissue Directives (EUCTDs) (2004/23/CE, 2006/17/CE and 2006/86/CE). These directives were designed to assure harmonized and high standards of Quality and Safety (QS) for the donation, procurement, testing, processing, preservation, storage and distribution of human cells and tissues, to facilitate their cross-border movements and to ensure availability in the EU. In 2007, the EUCTDs were supplemented with a regulation on Advanced Therapy Medicinal Products (ATMPs) (EC 1394/2007), including human tissue engineered and human somatic cell products, with additional requirements. This regulation should allow free movement of ATMPs within the EU market, better patients’ access to ATMPs, the highest level of health protection for patients, EU competitiveness in a key biotechnology area and growth of an emerging industry. These advanced therapies will transform treatment and prognosis of a number of diseases (e.g. myocardial infarct and Alzheimer) and thus hold huge potential for both patients and the industry. In the ATMP field, the major players are not large pharmaceutical companies, but rather small and medium-sized enterprises or (university) hospitals. With the EUCTDs and the ATMP regulation the EC did, however, introduce a series of expensive requirements and pharmaceutical industry standards, like Quality Management System (QMS) and guidance similar to Good Manufacturing Practices (GMP), into the field of HCT/P transplantation. For HCT/Ps that are classified as ATMPs, full blown GMP—which implies major investment in upgrading manufacturing facilities—is imposed. Suddenly the HCT/P field is confronted with practices and systems previously only required in pharmaceutical manufacturing.

## Cross-border movements

The EUCTDs were meant to facilitate cross-border movements of HCT/Ps. The heterogeneous transposition of the EUCTDs into EU Member State laws resulted in a patchwork of technical standards. At first sight, a setting that will not facilitate transnational movements. However, the unequal distribution of wealth and the lack of a global ethical framework (Pirnay et al. 2010) seem to create exploitation opportunities that are considered by some as unethical and solely based on profit-maximizing (cross-border) movements of HCT/Ps. Surely, where you have different regulations, you will have trading across the borders. Some TEs even considered using the international shipping legislation to bypass divergent national HCT/P regulations. For example, the world’s largest sperm bank explored the deployment of so-called ‘sperm ships’ flying Danish flags in the international waters based just outside the UK border to (legally) circumvent the strict UK In Vitro Fertilization (IVF) regulation (Hunter and Oultram 2008).

Unfortunately, cross-border movements of HCT/Ps strictly for (altruistic) medical reasons—as supposed to be facilitated via the EUCTDs—are still extremely rare.

## Raw materials

The EUCTDs were meant to secure safe procurement of human cells and tissues across Europe. In addition, the ATMP regulation intended to introduce certain requirements for manufacturing. However, it is clear that HCT/Ps are not only characterized by their manufacture, but also by their source, which gives rise to complex issues that are unusual for (ATMP) regulators and inspectors used to handling conventional (pharmaceutical) source materials (Farrugia 2000).

The main caveat is related to the limited supply of starting materials (donors) in respect of all types of human cells and tissues. This creates problems in view of companies who are put under pressure to maximize their profits as much as possible. For example, according to CNN Money, the product Alloderm™ (a skin substitute derived from human cadaveric skin) earned LifeCell the 16th place on FORTUNE’s 100 Fastest-Growing Companies list in 2004 and prompted them to recommend LifeCell’s stock (Birger 2006). One potential hitch was reported: raw material (human donor skin) supply constraints. In addition, there are certain unethical practices by some companies that try to get increasing amounts of raw tissues, preferably at low cost. International brokers and unprofessional middlemen are known to supply human organs, cells and tissues, obtained in low-income countries without self-sufficiency, basically located in Africa, Asia, Eastern Europe and South America, to the powerful industry in human tissues (Henkel 1994; Spurgeon 2008; AFSSAPS Alert Department 2008; Council of Europe 2009; Keller and Grill 2009). In this way, certain TEs in rich western European, North American and Asian countries obtain large amounts of raw materials for small fees, which in turn make welcome additions to salaries in countries with low salary levels. Supporters of these practices claim that these fees are used to develop the health care systems in these low-income countries. However, there are indications that in some cases these fees were transferred directly to the personal accounts of middlemen. The local health care system mostly remains deprived of the transplantation of the exported types of tissues. Apart from ethical and in some cases legal problems, these activities have caused major risks of transmission of diseases. An example happened in France in the late 80s. While the US and the UK halted the distribution of human growth hormone (in 1985) after it was discovered that people had died due to Creutzfeldt-Jakob disease after being treated with the product, which at that time was extracted from pituitary glands removed from corpses, doctors in France continued to use the hormone for several years, treating thousands of children. Nearly 60 % of the deaths worldwide caused by that treatment were in France. The media raised a thorny issue. Part of the pituitary glands were removed outside France, in Bulgaria, allegedly through a network of non-medical staff that took the pituitary glands from corpses in morgues for cash payments of a few francs per gland. According to media reports, the glands were often removed with crude instruments, such as coat hangers, through the nostrils of the corpse. As a result, contaminated brain tissue was sometimes also taken with the gland and was present in the extracted growth hormone (Spurgeon 2008).

In contrast to organs, there is no scarcity in tissues, at least in general terms. Tissue shortage is mostly due to organizational problems and/or a lack of human and material resources (Council of Europe 2009). What is then the answer to the key question ‘why do some TEs in rich countries prefer to procure human cells and tissues in developing countries?’ Are regulatory requirements in developing countries less stringent, procurement costs lower, rights of donor families less founded, or corruption in healthcare more widespread?

## Processing fees

The EUCTDs aimed at regulating the processing of human cells and tissues at a European level. In the EU (as is the case in the US as well) it is illegal to buy and sell human cells and tissues, even if they are procured outside Europe. The principle that it is not permissible for the human body or its parts as such to give rise to financial gain was established in Article 21 of the 1997 *Council of Europe Convention of Human Rights and Biomedicine*. Nevertheless, human cells and tissues are sold across borders worldwide, as it is not illegal to compensate hospitals, coroners and morgues for reasonable costs and charge ‘reasonable fees’ for the processing rather than the direct purchase of human cells and tissues. As the term ‘reasonable fee’ has not been defined, there is a grey zone and plenty room for misuse in terms of profit making from the processing of cells and tissues. In 2007, US Senator Charles Schumer introduced the *Safe Tissue Act*, designed to ‘improve the oversight and regulation of tissue banks and the tissue donation process, and for other purposes’ (Schumer 2007). The bill, if accepted, would determine the concept ‘reasonable processing fee.’ So far, the bill did not become law.

## Availability and patients’ access

The ultimate aim of the EUCTDs is to ensure the availability and patient’s access to HCT/Ps and there are no indications that it will not do that. But one must wonder (1) which HCT/Ps will mainly become available—the highly profitable or the medically important?—and (2) to whom will they be available—to everyone or only to those who can afford them?

### Highly profitable or medically important?

The interests of the general public, hospitals and corporate TEs are not always in line with each other and in certain cases they might be conflicting. Where hospitals mostly focus on medically important trajectories for health care, private TEs take a business approach to ensure their profits (for further investments and shareholders’ contributions), often taking a more lucrative approach with respect to the processing of donated cells or grafts. This is not because they are ‘bad.’ Under Anglo-American law, corporate managers even have a strict fiduciary duty to act in the interest of share-holders (Norman 2000). Examples of profit-maximizing activities are the systematic processing of human donor skin, the golden standard in the management of severe burns (Hermans 2011), into more lucrative products that can be used in plastic surgery or in vanity procedures such as penis-widening or lip enhancement in people with normal penis and lip sizes (Terino 1998; Bruno et al. 2007). More problematic is the possibility that some less lucrative, but life-saving, HCT/Ps will no longer be available. For example, in burn wound patients, the ideal replacement for missing skin is skin itself (Imahara and Klein 2009). In the absence of sufficient amounts of autologous grafts (the patient’s own skin), human donor skin (from cadavers) is without any doubt the next best thing. To date, there are no biosynthetic skin replacements that provide the physical and physiological functions of human skin. The signs are already there that industrially prepared biosynthetic dressings will replace human donor skin for the temporary covering of burns. Indeed, biosynthetic dressings are business as usual for pharmaceutical companies. They can be produced from widely available raw materials, which can be used in GMP production, and the resulting end products are standardized, well defined and—last but not least—can be adequately protected by patents. Human donor skin, as offered by conventional tissue banks, is a whole different ball game. The starting material is of variable ‘not standardized’ quality and inherently contaminated (at least with commensal bacteria) and the end product is also whimsical and—as ‘product of nature’—difficult to protect by patents today (Akst 2010).

Till recently, HCT/Ps produced and used at hospitals and not processed on an industrial basis, hence not aimed to be placed on the global market, were not considered as medicinal products. Today, the implementation of the ATMP regulation seems to ruffle feathers in the whole HCT/P landscape, illustrated by the following example. Keratinocytes produced by the keratinocyte bank of the Queen Astrid Military Hospital in Brussels have been used as auto- and allografts in more than 1,000 patients, primarily to accelerate the healing of burns and donor sites (De Corte et al. 2012). Since its creation in 1987, the keratinocyte bank has always been compliant with the relevant Belgian and European legislation and is licensed upon inspection by the competent authority. Since 2008, an ISO 9001 certified Quality Management System (QMS) governs all aspects of testing, processing, distribution, validation and traceability. Recently, the Committee for Advanced Therapies (CAT) of the European Medicines Agency (EMA) classified keratinocyte grafts as ATMPs. Full compliance with the ATMP regulation (without hospital exemption) would imply a dramatic and unbearable increase in production cost for the hospital at stake to offer this therapy. First of all, even if there could be a possibility to get a much higher reimbursement price within the national health system, responsible health practitioners would feel that keratinocytes in burn wound surgery do not warrant such an unnecessary high price. It would be an uncomfortable situation. Besides the purely economic aspects, public cell and tissue banks, like the hospital ones, are not necessarily interested in general market placement, centralized marketing authorization or intellectual property (IP) protection. Finally, the change in GMP requests will not necessarily lead to a measurable improvement of the QS of the keratinocyte grafts. The numerous inspections by the competent authorities at the hospital in the past 25 years never revealed the slightest health care risk. Conversely, for the industry, the potential market (severely burnt patients) is probably too small to consider. In practice, this means that in the course of 2012 (end of the transitional period for somatic cell ATMPs in Belgium), keratinocyte therapy, which has shown its usefulness in the past, will probably no longer be available to the severely burnt patients in the burn wound centers in Belgium.

### Available to everyone?

The development of HCT/Ps requests high investment costs if such products are aimed to be placed ‘on the market’ because for ATMPs, stringent and long-lasting regulatory procedures need to be complied with. The reimbursement of medical costs differs from country to country. In Belgium, health care insurance is part of the social security system. Medical costs are reimbursed by a health insurance fund and reimbursement rates are fixed by the government. The reimbursement rates of ‘conventional’ HCT/Ps are published in a ministerial decree (Federaal Agentschap voor Geneesmiddelen en Gezondheidsproducten 2009) that also fixes the price of lyophilization and WHO-approved prion- and virus-inactivation techniques. This price system for HCT/Ps is unique in the EU and was installed to cover the real costs of processing and to leave no room for unreasonable profits. In 2011, a Belgian stock market listed biomedical company received the notification by the Belgian Minister of Social Affairs of the approval of a convention agreement between the Belgian reimbursement authority for the reimbursement (for a period of 3 years) of ChondroCelect^®^, *characterized* autologous chondrocytes for the treatment of symptomatic knee cartilage lesions in well-indicated patients in specialized centers. Today, ChondroCelect^®^ is not only the first cell-based product to have obtained centralized European Marketing Authorization from EMA, it is also the first ATMP to obtain a national reimbursement (TiGenix 2011).

The reimbursement price (19,837 EUR for one application, without operation- and hospital costs) for ChondroCelect^®^ is nearly ten times the price of *conventional* non-ATMP and non-EMA approved autologous chondrocyte cultures (2,117.29 EUR for one application) in Belgium. Due to the high costs, the reimbursement of the procedure will be restricted to patients younger than 50 years.

The company’s clinical stage development pipeline includes an allogeneic stem cell product for the treatment of rheumatoid arthritis, a growing pharmaceutical market.

The reimbursement of this first approved ATMP in Europe to only a part of the potential patients indicates that social security systems will probably not be able to cope with the cost of future ATMPs. Who will then pay for these emerging therapies? The patients, whether or not through private insurances? When policy makers stated that the HCT/P legislation was installed to ensure patient’s access to HCT/Ps, probably they overlooked that not all patients could be served. Once an HCT/P is developed and approved, the pressure on companies and authorities to provide reimbursement becomes harmfully high. Therefore, the industry and the reimbursement authorities should decide which ATMPs will warrant future reimbursement (for every needy patient) and this prior to their development, which is often co-funded with tax money (the EU and most National funding agencies prioritize health research in support of industry). Biopharmaceuticals and biosimilars, other fast-growing segments of the pharmaceutical market, are also confronted with a risky, complex and expensive development process. Simoens (2009) suggested the early inclusion of health economics in the process of developing of biopharmaceuticals and biosimilars with a view to demonstrating their relative (cost) effectiveness and informing registration, pricing and reimbursement decisions.

One of the goals of the ATMP regulation was to ‘allow the highest level of health protection.’ How does a decrease (in number and in variety) of conventional grafts with well-established medicinal use and an increase in sophisticated commercially interesting products that are only accessible to a limited part of the population fit into this? Industry as well as the non-profit sector must reconsider how health care can be safeguarded for everyone in need for these therapies.

## Business techniques

Commercial companies raised the bar on processing technique. However, there are some companies that also introduced (extensive) marketing techniques, unreasonable (strategic) patenting activities and advertising efforts into cell and tissue banking. Marketing and advertising are known to have the power to influence consumer (physicians) habits and perceptions positively, but unfortunately also negatively. For example, some biomedical companies are known to promote the use of biosynthetic dressings. In (company driven) efficacy studies their product is compared with less efficient biosynthetic dressings at best (Rahmanian-Schwarz et al. 2011), never to the golden standard, human donor skin (Hermans 2011). There is a need for an independent assessment of the clinical relevance of newly developed therapies.

## Globalization

In 1983, Harvard Business School professor Theodore Levitt argued that companies should emphasize on offering standardized products all over the world (Levitt 1983). Companies that concentrated on idiosyncratic consumer preferences would not be able to take in the forest because of the trees. As today’s successful global brands demonstrate, this notion clearly makes sense from a linear/mechanistic economical point of view. As most (if not all) markets, the emerging global HCT/P market is inherently confronted with financial considerations. The current HCT/P legislations exhibit loopholes that allow excessively free maneuvering of those that seek economic advantage, which is quite logic from an economical point of view. And was it not one of the goals of the ATMP regulation to ‘allow competitiveness in a key biotechnology area and growth of an emerging industry’? Unfortunately, often service to the public health is not seen as a key priority. In the 1970s, most supporters of market economy embraced Friedman’s view (1970) that the social responsibility of business is to increase its profits, not to relax the conditions of profit-maximization on behalf of the wider interests of society. But, is this acceptable when it comes to healthcare? Surely, companies involved in the healthcare industry should live up to their responsibilities towards the public interest, not only towards their shareholders. To quote Bela Blasszauer (1997): ‘medicine is a moral enterprise whether it is practiced in the system of slavery or market economy.’

Defenders of Friedman’s thesis claim that for executives to use company resources to advance social goals would be for them to usurp the political function (Norman 2000). In this context it might thus be up to the political world to demand healthcare companies to defy the laws of economics and fulfill social duties.

## Conventional cell and tissue banks

The EC introduced industry standards for product development and marketing. However, these marketing activities often surpass their goals, in a field that was formerly dominated by altruistic hospital-based tissue banks. These banks are often not interested in the (global) marketing of their grafts and lack the regulatory experience and finances to implement the imposed requirements in due time. If the current evolutions in the field continue, in the near future European conventional cell and tissue banks will either throw in the towel or be reduced to facades (suppliers) for corporate TEs, especially where the cells and tissues are the basis for lucrative ATMPs.

One cause of the increased regulatory oversights was that, in the past, some tissue banks were indeed nonchalant in dealing with QS. However, there is a need for a sense of proportion and to make sure that the baby is not thrown out with the bath water. In his keynote speech at the 6th World Congress of Tissue Banks, Kearney (2011) explained that there is a need for public cell and tissue banks. They are, for example, far more efficient in the procurement of human tissues and turning them into natural matrices that can be re-populated by the patients’ own cells. The pharmaceutical industry, on the other hand, is far more efficient in the large-scale production of synthetic scaffolds and cell lines. It is key that public cell and tissue banks survive the introduction of expensive production and marketing requirements. A way for them to survive globalization could be to organize themselves in central tissue banks, which operate on a large scale (de Kort and Verhagen 2008), eventually partially sponsored by the government.

We fear that the implementation of the EU HCT/P legislation will ultimately lead to a globalized market with corporate TEs that will produce only a limited number of uniform HCT/Ps.

## Comparison with the food sector

There are striking parallels with the food sector. The rising liberalization of agro-industrial markets was also accompanied by technological advances and the introduction of an EU regulatory framework. In January 2006, the EU General Food Law (EC 178/2002) entered into force, introducing General Principles like GMP, Good Agricultural Practices (GAP), Good Distribution Practices (GDP) and requirements for traceability, responsibility and withdrawal in the food sector. In addition, the EU Hygiene Package (EC 852-854/2004) introduced further requirements such as registration, labeling, documentation and self-inspection. Small food producers, unable or not willing to go along with technological advances and new ideologies in marketing, are suffering under the new product safety regulations. Established (some are around for centuries) and tasty local products are suddenly presumed of inferior QS and are gradually replaced by uniform pale global brands, with (a perception of) high QS. Bioengineering is rapidly transforming the crop development industry, accelerating the concentration and centralization of agro-chemical corporations pushing (genetically modified) monocultures and undermining the cultural diversity of local farmers (McMichael 2001). Over the last decades, small independent beer brewers are diminishing in significance as brewing multinationals, resulting from mergers and (aggressive) acquisitions (Hurt 2010), have transformed one of the oldest industries in the world from a local market into a global one. Recently, the US artisan cheese world was shaken by the shutdown, by the US Federal Drug Agency (FDA), of several small (award-winning) cheese making facilities, due to bacteria findings in cheeses (Perlman 2011). Those defending the age-old methods of local craftsmen find the QS rules and inspections to be over the top and argue that the products of large-scale food companies have caused many more illnesses than any product from small producers. Of course, in the future some fine specialties will still be produced and globally distributed, according to the new requirements, as delicacies (e.g. French ‘Grand Cru’ wines) for people who can afford it.

## Commodification

A scene in the 1973 movie ‘Soylent Green’ comes to mind. It is the year 2022 and, as natural resources have been exhausted, people are fed synthetic Soylent products (green crackers said to be made of plankton). Detective Thorn (Charlton Heston) steals a number of food items from the home of a wealthy murder victim. Although they used to be everyday foods like wine, apples and meat, his older friend Sol Roth, who remembers the time when tasty food was plenty, breaks down into tears at the thought of meat. At the end of the movie Thorn uncovers the disturbing truth about the real ingredients of Soylent Green, recycled human bodies. But, why was this discovery so disturbing and even appalling to viewers? In times of overpopulation, famine and no resources, human bodies *do* constitute a vital source of food. Chimpanzees’ need to feed sometimes leads them to cannibalism. Moreover, in the movie, it surely looks like Soylent Green is produced according to high QS requirements. So, why should the optimum utilization of human bodies not be explored? Probably because our civilization, for centuries, accepts and demands respect for the dead (Marcus 1985). Turning human bodies (in secret) into lucrative products for a global food (or pharmaceutical) industry would not be very respectful. Critics of markets in body parts state that they are ethically wrong because they violate a fundamental ethical norm that the body should not be treated either as a property or as a commodity (Council of Europe 2009). Donor families expect HCT/Ps to be treated with respect and recognized as resulting from a donation from their loved ones (Yessian 2001). Instead, tissues donated to tissue banks are increasingly processed into *products* with little or no resemblance to human tissue like cubes, screws, chips, paste, glue and powder, which are then sealed in appealing packaging and advertised in glossy catalogs as if they were commodities. Thorn’s final warning ‘Soon they’ll be breeding us like cattle’ points at another possible abomination of the commodification of human bodies.

## Quality and safety requirements

Most incidents involving unsafe HCT/Ps were not the result of too loose QS requirements in legislations. They were due to the greed of opportunists that downright ignored the guidelines and common sense and engaged in profit-maximizing activities that ultimately endangered patients and trampled ethics. Importantly, these incidents are not representative of the entire tissue banking community. In Belgium, as in most EU Member States, the national pre-EUCTD human cell and tissue legislations and quality standards functioned well. They succeeded in safeguarding the provision of acceptable amounts of affordable, safe and ethically sound HCT/Ps.

There is no doubt that the implementation of the EU HCT/P legislation will increase overall QS to the HCT/P field. However, we have to keep in mind that QS is no fairy dust or magic formula. A false perception of QS is creeping in. For example, recently, the French authorities issued a guideline urging 30,000 French women to have their breast implants removed (Chrisafis 2011). A French company was found to be cutting corners by making breast implants from cheaper industrial-grade silicone normally used for electronics, mattresses or the agriculture industry. In addition, these implants have a relatively high chance of bursting. And yet, they were granted a certificate of conformity with European standards and hundreds of thousands of them were sold on three continents.

How do regulations see to improve the measurable benefits to patient care and safety, taking into account the considerable burdens on service providers and businesses, and ultimately the community as a whole (Kirkland 2010)? In some cases, the substantial increase in requirements introduced by the EUCTDs and the ATMP regulation result in a massive increase in costs (material and personnel), without measurable gain in QS.

Unfortunately, today, HCT/Ps seem to be regulated through manufacturing assessment and any issues of therapeutic efficacy or benefit to the patient are side-stepped (Farrugia 2000). What does the concept ‘Quality and Safety’ really mean? According to WHO Europe guidance (2008), ‘a quality health service is one which organizes resources in the most effective way to meet the health needs of those most in need, for prevention and care, safely, without waste and within higher level requirements’. It is our feeling that when it comes to the EUCTDs, policy makers limited the definition of QS to ‘safe and within higher level requirements.’ Strangely, ‘quality’ and ‘safety’ are *always* pronounced in one breath and seem to be reduced to synonyms. In addition, we get the feeling that ‘safe’ almost exclusively means ‘free of transmissible diseases.’ There is nothing in the EUCTDs that prescribes that prepared HCT/Ps must be of high quality and safe in a sense of achieving the intended clinical utility (van Veen and Lamers 2006). For example, commercial autologous cord blood banks are emerging worldwide. Some of them take advantage of the vulnerability and ignorance of new parents to urge them to store the cord blood for ‘possible’ future clinical use in their child, its siblings or family members. For this service they charge handsome fees (2,395 EUR in Belgium). There are, however, no indications that these autologous stem cells will be more effective than allogeneic stem cells stored in public cord banks and are accessible to all patients in need (Brand et al. 2008).

In our opinion, the safest HCT/P is not necessarily the most qualitative or the most effective way to meet health needs of those most in need. There is a point at which legislation can actually compromise patient care and safety, by hindering valuable established therapies or delaying the development of new technologies. Efficacy should not be sacrificed in the name of QS. In the end, what saves more severely burnt patients’ lives, conventional human donor skin with an infinite small risk of disease transmission or sterile biosynthetic dressings? According to the current generation of surgeons in the burn wound center of the Military Hospital, without any doubt, the former.

In addition, the EUCTD QS requirements are generic (not tailored to specific HCT/Ps). As such, they apply to heart valves as well as to skin. Heart valves are sterile at the time of harvesting and will be grafted internally during an aseptic surgical procedure in an operating theatre. Skin, in contrast, is inhabited with micro-organisms (commensals), which out-compete potentially harmful bacteria and prevent them from inhabiting the skin surface. In addition, the skin of donors is in contact with the (uncontrolled) environment during the entire life of the donor. Upon death, the non-heart beating skin donor is kept in a fridge in the mortuary for many hours before skin procurement takes place. The harvested donor skin is applied in a hydrotherapy facility, a room in the 1-day clinic or an operating theatre at best, where it is grafted on the surface of non-sterile and often infected burn wounds next to the patient’s intact skin that is colonized with commensals. Yet, the same arbitrary clean room air quality requirements for tissue processing (which only takes a few hours), should be applied to heart valves and skin alike. For IVF laboratories, these air quality requirements will not only have a negligible impact on QS, they will probably compromise the ability to maintain gametes and embryos under optimum environmental conditions (Mortimer 2005).

While some QS requirements are based on objective evidence, others seem to have been whispered by the precautionary principle, a key paradigm of current regulatory thinking. This principle can be expressed as: ‘complete evidence of risk does not have to exist to institute measures to protect individuals and society from that risk.’ According to Kirkland (2010), we should try to balance the risk avoidance principles with the broader risks to the community that can result from overzealous or inappropriate application of regulatory standards (e.g. consider the access to life-saving therapies with a certain risk of disease transmission). This can only happen if the application of these regulations is flexible, adaptable and subject to review. Within an EC co-funded project, rational and tissue specific European Good Tissue Practices (EuroGTPs) are being developed (www.euroGTPs.com). It is not sure whether these EuroGTPs will have binding power in the (near) future.

## Enforcement

Bone donations to a Bulgarian tissue bank are sent to a TE in New Jersey USA for processing. The finished products are sent to TEs in more than 20 countries in 4 continents, including a TE in France. In 2008, a joint inspection was conducted by AFSSAPS, the French competent authority, and the Bulgarian Executive Agency. The inspection highlighted one critical and 10 major deficiencies that were not in compliance with the EUCTDs. These deficiencies were related to procurement activities. There were serious concerns regarding traceability and validity of blood sample labeling and donor records. AFSSAPS requested the recall of bone products supplied by the French TE (AFSSAPS Alert Department 2008).

Unfortunately, such thorough cross-border and human cell and tissue procurement site inspections are only rarely performed. In addition, competent authorities’ inspectors often lack the expertise, guidance, training (e.g. collected evidence must be relevant for use in court) and power (compared to police) to swiftly and efficiently act against Illegal or Fraudulent Activity (IFA) as it is called today. The enforcement of the EU HCT/P legislation should become more efficient (Kishore 2005). Therefore, in March 2010, the EU-funded project ‘Vigilance and surveillance of substances of human origin’ (SOHO V&S) was launched (Fehily 2011).

Obviously, the increasing number of Legal Excessive Profit-making Activities (LEPRAs) cannot be countered by more efficient and more frequent inspections. Limiting the profit that can be made on the processing and resale of HCT/Ps, for example by fixing prices, would at least remove the incentive for LEPRAs.

## Discussion and conclusions

In 1985, author and philosopher Malcolm Muggeridge warned in his key note address at an international symposium on organs and transplantation held at Lake Louise (Canada) that the ‘hacking out of bits of peoples organs and putting them on the market is becoming an extraordinarily lucrative occupation. It’s going to be a very big trade’ and ‘where you have money being the decisive factor, there you will have trouble and disruption inevitably’ (Marcus 1985). More than a quarter of a century later the ‘declaration of Istanbul on organ trafficking and transplant tourism’ urges EU Member States ‘to take measures to protect the poorest and vulnerable groups for transplant tourism and the sale of tissues and organs, including attention to the wider problem of international trafficking in human tissues and organs’ (Steering Committee of the Istanbul Summit 2008). Today, Malcolm Muggeridge’s forecast has thus come true as a gradual model of commercialization and commodification of human cells and tissues can be observed, also in Europe, where healthcare is increasingly governed by EU legislation. The EUCTDs are enacted through common QS standards and evade public debate because they are merely seen as ‘technical matters’ (Hoeyer 2010). Yet, the implementations of the EUCTDs that appear to be necessary to policy makers, while at the same time remaining somewhat disconnected from the everyday reality of cell and tissue bankers, hold serious dangers, which need to be urgently addressed.

The current EU HCT/P regulatory framework allows for-profit TEs and facilitates the development of a uniform global HCT/P market, and is not able to deal with the technological innovation and controversial market-driven practices that raise deep ethical issues today.

As a result, the most profitable HCT/Ps are the ones that are most likely to be developed in the interest of shareholders, which takes precedence over the public interests. In addition, it is questionable whether the health care and social security systems of EU Member States will be able to cope with the rising health care costs entailed by increasingly stringent QS requirements.

Why is the regulatory framework not able to curb this? First of all, ethical issues (e.g. allocation rules) could not be addressed as the EC was not mandated by the Maastricht Treaty (that lead to the creation of the European Union) to do so. For organs, allocation issues have been addressed by a number of not-for-profit service organizations like Eurotransplant International Foundation, Scandiatransplant, and the National Health Service Blood and Transplant in the UK.

Secondly, while there were numerous concerns in the cell and tissue banking world (e.g. LEPRAs were already emerging), the minimization of infectious disease risk turned out to be the paramount driver for the introduction of more regulation in this field.

Finally, Lenk and Beier (2012) recently argued that the ban on commercialization of body material is not as strict as it may appear at first sight, leaving room for commercial practice of tissue procurement and transfer. On the one hand EU policy makers claim they wish to avoid the commercialization of HCT/Ps, but on the other hand they are apparently very reluctant to put this into hard (binding) wording. According to Faulkner et al. (2006), the EUCTD was created through a democratic process and professional trade associations such as EUCOMED and EuropaBio lobbied extensively on this regulation. It is logic for a business to ward off profit-reducing regulation. Public altruistic cell and tissue banks simply lacked organization, power and experience to lobby relevant EU legislation.

There are, however, extenuating circumstances. When the EU HCT/P legislation was developed, in the late 90s—early 2000s, the HCT/P transplantation field was in its infancy. But, it rapidly grew from a ‘cottage industry’ of small non-profit and predominantly hospital-based surgical banks, which provided minimally processed tissues to local surgeons, to a booming industry in which highly sophisticated HCT/Ps are distributed worldwide. The field has become much more complex, with technical advances and extensive commercialization, than the policy makers and experts expected when they started elaborating the EUCTDs. In addition, there are indications that key aspects of European policies designed to protect public health were undermined in a generic way by certain players in the field. Recently, it was demonstrated that from 1995 an alliance of corporate actors actively worked to successfully promote a business-oriented form of Impact Assessment (IA) of *all* major EU policies (Smith et al. 2010). This increases the likelihood that the EU produces policies that advance the interests of major corporations, including those that produce products damaging health, rather than in the interest of its citizens. A health-oriented IA involving all stakeholders would have been more appropriate in assessing public health policies.

It’s about time for a comprehensive review and revision of the EU HCT/P regulation. This poses an acute policy maker’s dilemma. Some feel that the commercialization and commodification of HCT/Ps should be restricted, in the name of the overall public health framework, including patients and donor families. On the other hand, pharmaceutical and biomedical companies are no charitable organizations; their goal is to maximize shareholders’ profits. Should the European biotechnology be denied a commercial opportunity? Politicians are not immune to these tensions. It must also be said that HCT/P commercialization issues are complex and need to be dealt with differentially. In some cases commercialization of human cells and tissues should be avoided and HCT/Ps should be provided by public institutions at fixed (e.g. by the government) reimbursement (cost recovery) rates. For some sophisticated HCT/Ps, reasonable processing fees could be tolerated to offer incentives to (tissue engineering) companies. Controversial LEPRAs, however, should be banned altogether.

Cynics believe that the commercialization of all aspects of society is inevitable and resistance futile. However, if EU policy makers decide to give priority to the overall public interest and halt the erosion of public healthcare systems, they should update the HCT/P legislation to:

- prioritize the solidarity principle of public TEs;
- prioritize medically relevant HCT/Ps;
- introduce cell or tissue specific QS requirements based on common sense and objective evidence;
- control HCT/P prices through a regulatory mechanism;
- introduce fair, transparent and binding exportation rules with an emphasis on self-sufficiency;
- be enforced by efficient (cross border) inspections.

On the other hand, if the globalization of the healthcare industry is part of a political philosophy and EU policy makers decide to continue on the route of HCT/P commercialization, they should clearly speak their mind. Under the pretext of food safety, legislation facilitated the concentration, or even globalization, of the food supply. Is the HCT/P transplantation field about to drift in the same direction? Are QS requirements like GMP becoming selection pressures for HCT/Ps?

In an interview with the New York Times (Blakeslee 2002) Theodore Malinin of the University of Miami Tissue Bank stated: ‘tissue donation is an altruistic act and it may be incompatible with the desire to make money.’
